# First molecular confirmation of *Lasiodiplodia theobromae* causing grapevine trunk disease in southern Egypt

**DOI:** 10.1038/s41598-025-32284-4

**Published:** 2025-12-29

**Authors:** Youssuf A. Gherbawy, Nabila A. Hassany, Eman G. A. M. El‐Dawy

**Affiliations:** 1 Department of Botany and Microbiology, Faculty of Science, South Valley University (Qena University), Qena, 83523 Egypt; 2 Applied and Environmental Microbiology Center, Faculty of Science, South Valley University (Qena University), Qena, 83523 Egypt

**Keywords:** *Lasiodiplodia theobromae*, Phylogeny, Pathogenicity test, Egyptian vineyards, Microbial ecology, Pathogens

## Abstract

Grapevine trunk diseases constitute a significant phytopathological concern in Egyptian viticulture, with ongoing debates regarding their origin and transmission dynamics. These complexities are attributed to the heterogeneous manifestation of symptoms and the involvement of multiple wood-associated pathogens, both suspected and confirmed. This study investigates the mycological aspects of grapevine trunk diseases, focusing on *Lasiodiplodia theobromae* as a causal agent. The pathogen was associated with vascular cankers, dark brown trunk discoloration, pycnidia formation on necrotic tissues, and grapevine dieback. Identification of *L. theobromae* was achieved through morphological characteristics and molecular analysis targeting the β-tubulin gene and Internal Transcribed Spacer (ITS) region. Pathogenicity tests were conducted by inoculating detached canes, leaves, petioles, and entire branches with mycelial plugs of *L. theobromae*. The resulting symptoms closely resembled those observed in naturally infected grapevines in the field. The pathogen was then re-isolated and identified, confirming Koch’s postulates. A disease index (DI) ranging from 60 to 100% provided strong evidence of the high pathogenic potential of *L. theobromae* under experimental conditions.

## Introduction

Grapevine trunk diseases (GTDs) are a complex group of disorders that affect the woody tissues of grapevines and pose a significant effect on viticulture worldwide, including in Egypt. The three main grapevine trunk diseases are Esca disease, Eutypa dieback, and Botryosphaeria dieback^1^. These diseases are linked to the presence of various fungi, including *Phaeomoniella chlamydospora*, *Phaeoacremonium minimum*, *Fomitiporia mediterranea*, and *Botryosphaeriaceae* species^2^, all of which contribute to the degradation of woody tissues. The progression of these diseases often leads to vine decline and dieback, primarily due to the colonization of vascular tissues and infection of the perennial organs by pathogenic fungi.

Fungal species belonging to the Botryosphaeriaceae family produce a progressive wood disease known as “Botryosphaeria dieback”, found worldwide and is more common in areas with warm temperatures^3^. *Lasiodiplodia* belongs to Botryosphaeriaceae; the main symptoms caused by this fungus are vascular cankers discoloration, extending to the death of plant branches and dieback^4^. *Botryodiplodia theobromae* Pat was first reported by El-Goorani and El Meleigi^5^ and revealed to cause canker, soft rot, and grapevine dieback. *L. theobromae* group is a cryptic species comprised of more than 13 species^6^.

This study aimed to characterize *L. theobromae* isolates from Egyptian vineyards through morphological and molecular analyses, and to evaluate their pathogenicity on grapevine tissues. Although *L. theobromae* has been reported as a grapevine pathogen in other regions, its occurrence, genetic profile, and virulence in Egyptian viticultural systems remain poorly documented. By addressing this gap, the study provides new insights into the local epidemiology and pathogenic behavior of *L. theobromae*, contributing to improved disease diagnosis and management strategies in Egypt.

## Materials and methods

### Samples collection and pathogens isolation

From 2022 to 2024, in Qena, Egypt, samples infected with GTD were collected. The trunk samples showed brown and black longitudinal streaks. Trunk samples from symptomatic plants were washed under tap water and dried. Small pieces of wood (5 mm^3^) were taken from the diseased tissues, submerged in 2.5% sodium hypochlorite for 2 min, washed twice in sterile distilled water, and dried with sterile filter papers. Wood pieces were plated on potato dextrose agar medium (PDA) and incubated at 28 °C for 5 days^7^**.** Fungal colonies were transferred to fresh PDA plates, and single spore cultures were purified after sporulation.

### Morphology of isolated pathogens

Colony characteristics were observed for ten isolates cultured on PDA medium at 28 °C for 7 days. All isolates exhibited similar morphological features, including the appearance of colonies and the structure of conidiogenous cells, paraphyses, and both immature and mature conidia. Based on this uniformity, one representative isolate was selected for detailed morphological characterization using an Olympus Microscope Digital Camera SC 180 at 40 × magnification. For accurate identification, sterile pieces of trunk tissues were cultured on PDA medium and inoculated with the same pathogen. Additionally, all ten isolates of *L. theobromae* were used in the pathogenicity tests to confirm their disease-causing ability.

### Molecular analysis

The pathogen was grown for 2 days at 28 °C on PDA medium (SRL). Genomic DNA was extracted using the 2 × cetyltrimethylammonium bromide buffer (CTAB) protocol as described by Moller et al.^8^. DNA quality was verified by electrophoresis on a 1.4% agarose gel stained with ethidium bromide and visualized under a UV transilluminator.

PCR amplification was performed targeting both the β-tubulin gene using Bt2a-Bt2b primers^9^ and the Internal Transcribed Spacer (ITS) region using universal ITS1 and ITS4 primers. PCR products were purified and sequenced at Macrogen, South Korea.

The obtained sequences were compared against the GenBank database using the BLASTn tool from the National Center for Biotechnology Information (NCBI). Phylogenetic analyses were conducted using a neighbor-joining algorithm with the maximum composite likelihood model implemented in TREECON Windows (version 1.3b, 1998)^10^. Bootstrap analysis was performed with 100 replicates to assess the robustness of the branching, and phylogenetic trees were constructed separately for β-tubulin and ITS sequences.

### Pathogenicity test

Pathogenicity test and re-isolation of the pathogen were conducted in accordance with Koch’s postulates. Fresh grapevine tissues, including canes, petioles, leaves, and whole branches, were used for inoculation. Each tissue type was first rinsed with tap water, then surface-sterilized using 5% sodium hypochlorite, and placed in sterile Petri dishes containing two filter papers. To maintain high humidity, 10 ml of sterile distilled water was added to each plate. An 8 mm disc was excised from the actively growing margin of a 7-day-old culture of *L. theobromae* and placed on the surface of sterilized grapevine tissues, including canes, petioles, and leaves. For the branch samples, a total of nine were used, with three replicates for each, and they were placed in sterile conical flasks containing distilled water to maintain humidity. All samples were incubated at 28 ± 2 °C, and lesion development was monitored after 7 days. Control samples received no fungal inoculum. The experiment was conducted in triplicate for each tissue type, and plates were incubated at 28 ± 2 °C. Lesion development was observed after 7 days [^11^].

Disease severity was quantified using the Disease Index (DI), calculated as^12^:

$${\mathrm{DI}}\;{\text{(\% )}} = \left( {\frac{{\sum \left( {{\mathrm{disease}}\;{\mathrm{score}} \times {\mathrm{number}}\;{\mathrm{of}}\;{\mathrm{sections}}} \right)}}{{{\mathrm{total}}\;{\mathrm{sections}} \times {\mathrm{maximum}}\;{\mathrm{score}}}}} \right) \times 100.$$

To assess differences in pathogenicity among tissue types, data were analyzed using SPSS version 20 (SPSS Inc., Chicago, IL, USA), one-way ANOVA followed by Least Significant Difference (LSD) tests at a significance level of p < 0.05.

## Results

### Symptoms of grapevine trunk disease

**Fig. 1:**
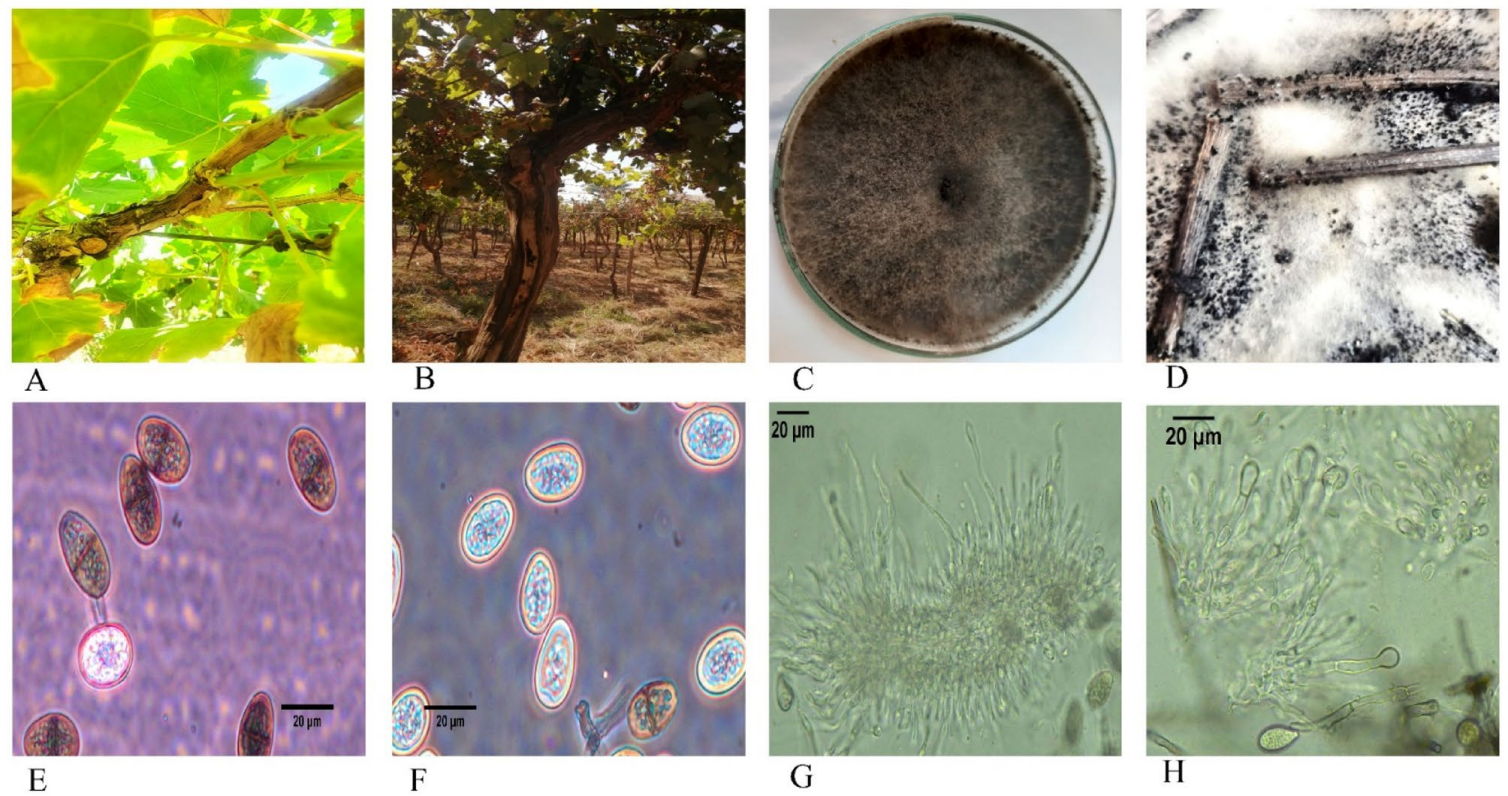
Infected plant (**A**, **B**) and morphological characteristics of *Lasiodiplodia theobromae* (OR995259): colony on PDA (**C**); Pycnidium (**D**); mature conidium (**E**); immature conidium (**F**); Conidiogenous cells and paraphyses (**G**, **H**). Magnification 40 × , Scale Bar = 20 µm.

From 2022 to 2024, diseased trunk tissues showed a dark brown color with streaks, leading to drying out and dieback. Dark brown to black pycnidia were found on the infected trunk tissues. (Fig. 1A, B).

### Morphological identification

In all collected samples, a common isolated species was *L. theobromae,* which appeared with white turned to dark olivaceous with dense aerial mycelium (Fig. 1C). Pycnidia appeared with black or dark brown formed on grapevine canes (Fig. 1D). Mature conidia showed dark brown with one septum; out of 40 conidia, the average size was (20.9–23.7 × 12.4–14.6 µm), produced initially hyaline and aseptate, sub ovoid to ellipsoid-ovoid, (19.5–20 × 13.3–13.6 µm) (Fig. 1E, F). Paraphyses were hyaline, cylindrical, and septate (Fig. 1G, H).

### Phylogeny analysis

Amplicons from both the ITS (PX122069) and β-tubulin (OR995259) regions of *Lasiodiplodia theobromae* were sequenced and deposited in GenBank. BLASTn analysis of these sequences showed 100% identity with *L. theobromae* reference entries, including OR551845, PQ479075, OL405583, OR551950, MW287589, ON787781, MW287591, MK570084, and others.

Phylogenetic trees based on partial ITS (panel A) and β-tubulin (panel B) gene sequences were constructed using the neighbor-joining algorithm with the maximum composite likelihood model. Bootstrap values from 100 replicates are indicated at the nodes, and values below 50% are not shown. The trees were rooted using *Alternaria abundans* (ITS: OL457279; β-tubulin: JQ671968) as outgroups.

**Fig. 2:**
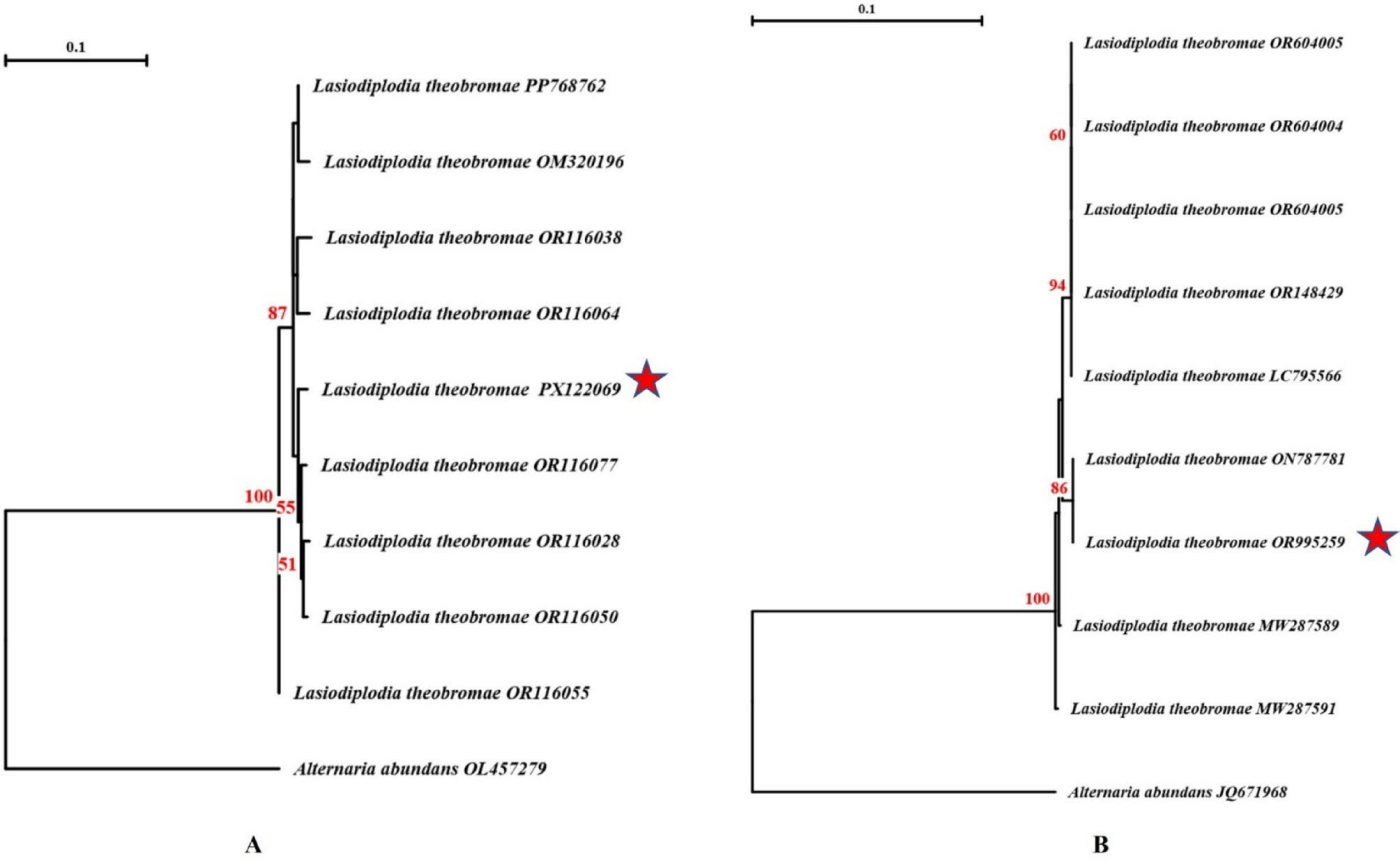
Phylogenetic trees based on partial ITS (**A**) and β-tubulin (**B**) gene sequences of *Lasiodiplodia theobromae* isolates (PX122069 and OR995259), constructed using the neighbor-joining algorithm with the maximum composite likelihood model. Bootstrap values from 100 replicates are shown at nodes (values < 50% omitted). *Alternaria abundans* was used as an outgroup (**A**: OL457279; **B**: JQ671968). Isolates from this study are marked with red asterisks (*).

Our isolates (*L. theobromae* PX122069 and OR995259), marked with red asterisks, clustered tightly with established *L. theobromae* sequences from GenBank, with high bootstrap support (up to 100%) confirming their taxonomic placement. These results unequivocally demonstrate the close evolutionary relationship of our isolates to known *L. theobromae* strains and provide robust molecular evidence supporting their identification (Fig. 2).

### Pathogenicity activity

**Fig. 3:**
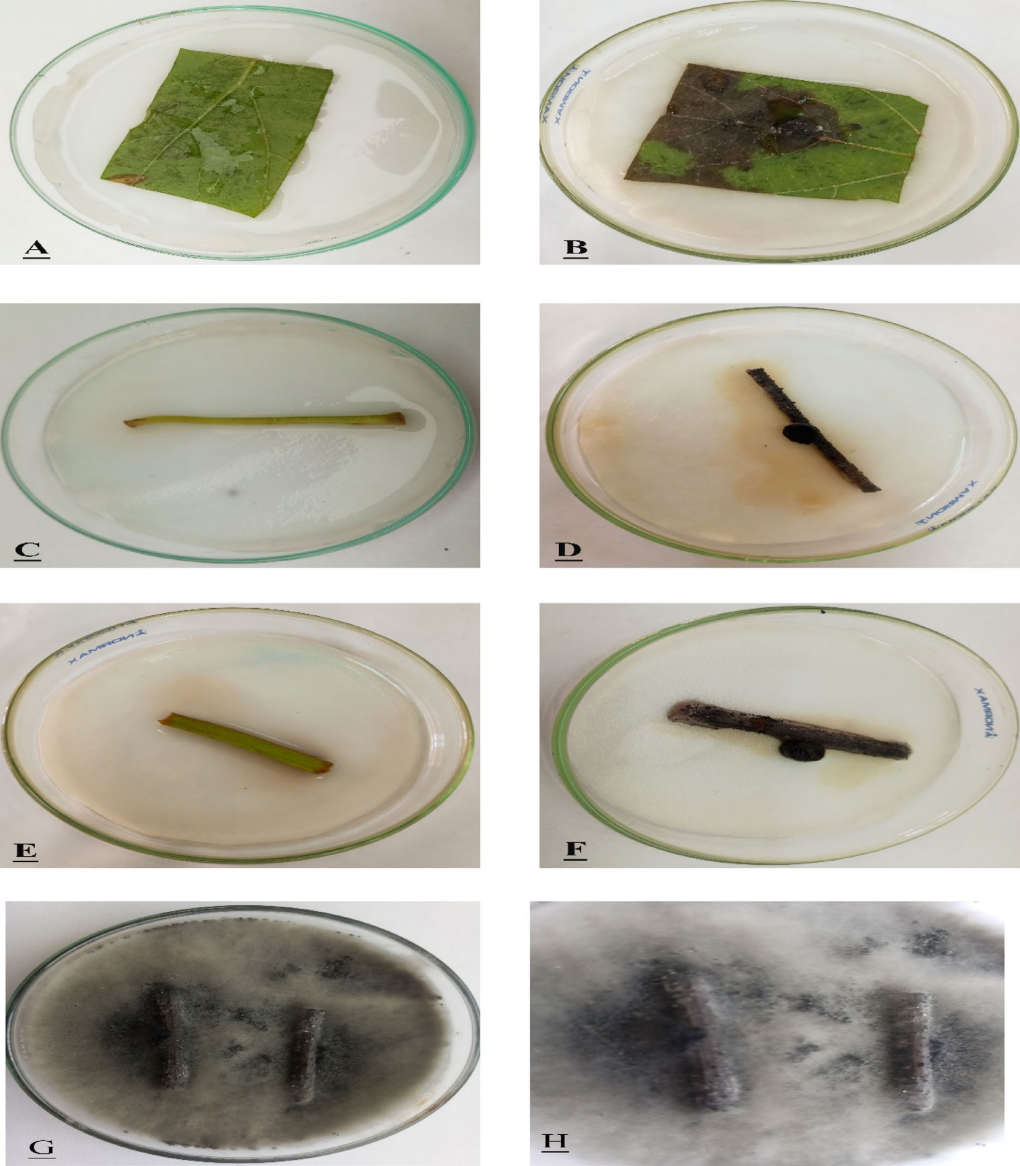
Pathogenicity effect of *Lasiodiplodia theobromae* (OR995259) on the leaves (**B**), petioles (**D**) and canes of grapevine (**F**), incubation at 28 °C, for 1 week. (**A**, **C**, **E**) Control. (**G**, **H**) Re-isolation of *L. theobromae* on PDA medium after 7 days at 28 °C.

**Fig. 4:**
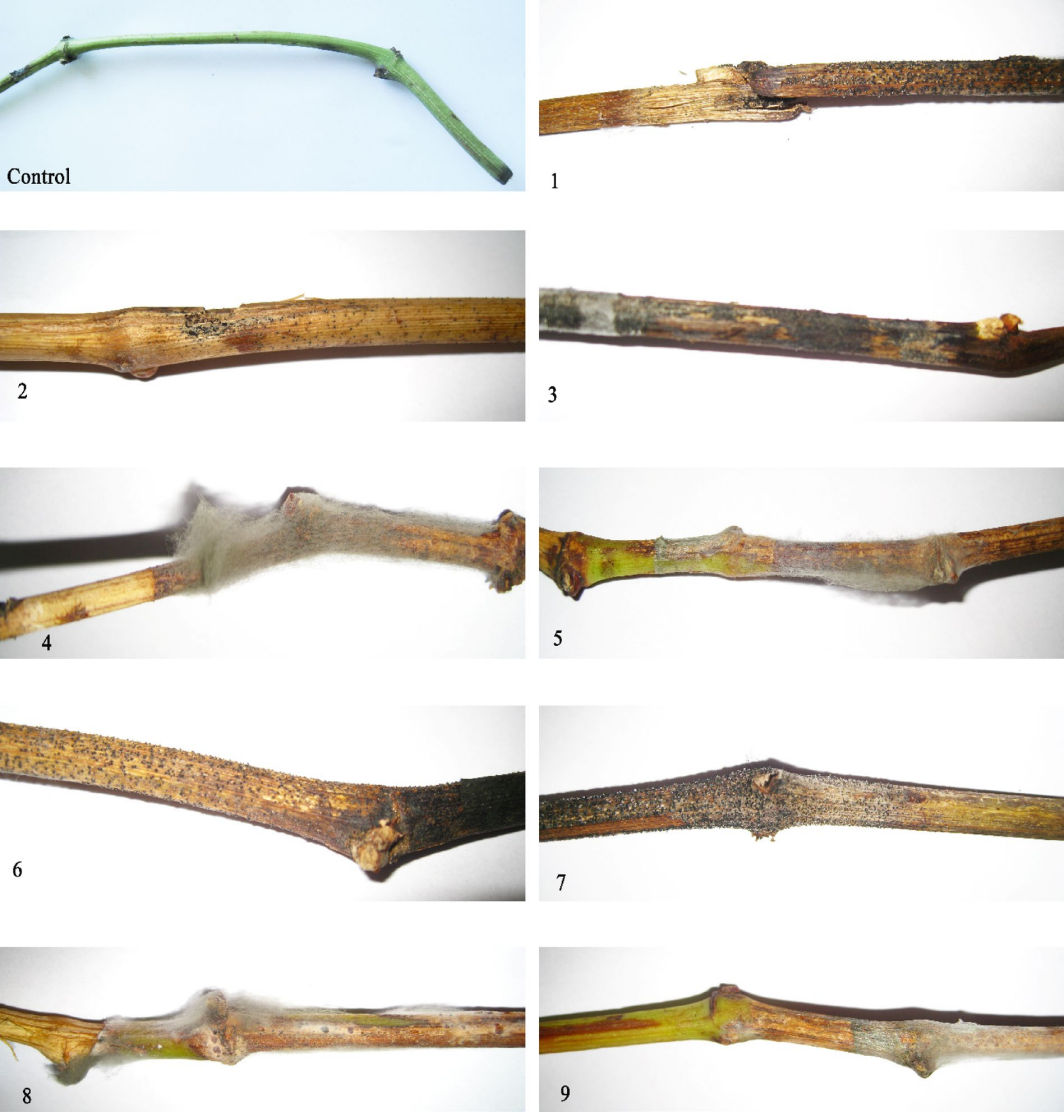
Severity of symptoms caused by *Lasiodiplodia theobromae* on grapevine branches following incubation at 28 °C for 1 week, using isolates 1 to 9.

To detect the pathogenicity activity of *L. theobromae*, inoculation was conducted on detached grapevine canes, leaves, petioles, and the whole branches. After 1 week, lesions of dark brown color appeared on the inoculated parts. Pycnidia were observed. The Disease Index (DI) values recorded for leaf, petiole, and cane tissues were 93.3%, 93.3%, and 100%, respectively, indicating a consistently high level of infection across all tissue types. Among the nine grapevine branches tested, five exhibited complete disease incidence (100% DI), three showed severe symptoms (80% DI), and one displayed moderate severity (60% DI). Statistical analysis confirmed that all DI values were significantly high, reflecting the strong pathogenic potential of isolates. The pathogen was successfully re-isolated from symptomatic tissues and exhibited identical cultural and morphological characteristics to the original inoculated strain (Figs. 3, 4, and Table 1).

**Table 1:** Disease index (DI) of grapevine tissues (petioles, cane, leaves, and branches) following inoculation with isolates of *Lasiodiplodia theobromae*, incubation at 28 °C for 1 week.

Type of tissue and isolate no	Disease index (DI)%
Leaf 1	93.3*
Petiole 1	93.3*
Cane 1	100*
Branch 1	100*
Branch 2	60*
Branch 3	100*
Branch 4	80*
Branch 5	80*
Branch 6	100*
Branch 7	100*
Branch 8	80*
Branch 9	100*

*Means of DI% was significant at *p* < 0.05.

## Discussion

This report confirms *Lasiodiplodia theobromae* as the causative agent of grapevine trunk disease (GTD) in Qena, Egypt, based on integrated morphological observations and comprehensive molecular analyses, including sequencing of the ITS and β-tubulin gene regions accompanied by phylogenetic tree construction. Our isolates exhibited 100% sequence identity with reference *L. theobromae* sequences in GenBank and clustered closely with established strains, providing compelling taxonomic confirmation. Úrbez-Torres et al.^13^ reported that the phylogeny analyses using three different gene regions (ITS, β-tubulin, and EF1-α) all produced the same overall tree structure. Both the combined and individual gene datasets confirmed the identity of *Lasiodiplodia theobromae* and *Diplodia seriata* found on grapevines in Mexico, matching with the morphological identification. This species was first identified in Egyptian vineyards as *Botryodiplodia theobromae* Pat^5^, and subsequent studies have established its continued presence and epidemiological significance^14^**.**

Internationally, *L. theobromae* is reported as a widespread pathogen of grapevine causing dieback in regions such as Iraq^15^ and Peru^7^, with phylogenetic evidence supporting its global dissemination. Literature points to considerable diversity and adaptability in *L. theobromae* isolates, reflected by variations in pathogenicity and host range^6,16,17^. Its prevalence is notably higher in warm climates, suggesting ecological adaptation that supports infection under local environmental stresses^4,18^. *L. theobromae* is a highly adaptable pathogen capable of infecting diverse plant tissues and surviving under various environmental stressors, which contributes to its widespread distribution and impact on perennial crops. Our study provides the first molecular evidence of *L. theobromae* infecting grapevines in this region of Egypt, a finding that is critical for accurate diagnosis and targeted disease management. Molecular characterization validates the pathogen’s identity while also laying the framework for monitoring its transmission and understanding its population structure. The pathogen’s ability to produce bioactive secondary metabolites and its temperature responsive gene expression, particularly genes involved in cell wall degradation and toxin production highlight the complexity of its pathogenicity. Understanding these molecular and environmental interactions is essential for developing effective management strategies. Given the high Disease Index values observed across all tested tissues, our findings underscore the urgent need for integrated disease management approaches. These may include the use of resistant grapevine cultivars, timely application of effective fungicides, and cultural practices that reduce plant stress and inoculum sources. Future research should also explore the genetic diversity of *L. theobromae* populations and evaluate the efficacy of biological control agents to support sustainable viticulture^19^.

Epidemiologically, our high Disease Index (DI) results echo those from El-Habbaa^14^, confirming its dominance in Egyptian vineyards and impact on grapevine health, with aggressive disease symptoms reflected in rapid tissue dieback and pycnidia formation. Comparisons with regional and international literature reinforce the need for ongoing surveillance and improved disease management strategies to mitigate losses, particularly as viticulture expands in Egypt’s southern regions.

Through detailed molecular characterization and pathogenicity assessment, our study enriches the epidemiological understanding of *L. theobromae* in Egypt. It underscores the importance of ongoing pathogen surveillance alongside the implementation of integrated disease control practices tailored to the local agroecological conditions.

## Data Availability

The sequence data generated and analysed during the current study are available in the NCBI GenBank repository, https://www.ncbi.nlm.nih.gov/nucleotide/. The accession no. of our isolate was OR995259 and PX122069, available in https://www.ncbi.nlm.nih.gov/nuccore/OR995259 and https://www.ncbi.nlm.nih.gov/search/all/?term=PX122069.
